# Ultrathin sulfate-intercalated NiFe-layered double hydroxide nanosheets for efficient electrocatalytic oxygen evolution†

**DOI:** 10.1039/d0ra00845a

**Published:** 2020-03-25

**Authors:** Xiao-Xiao Jiang, Jiang-Yan Xue, Zhong-Yin Zhao, Cong Li, Fei-Long Li, Chen Cao, Zheng Niu, Hong-Wei Gu, Jian-Ping Lang

**Affiliations:** College of Chemistry, Chemical Engineering and Materials Science, Soochow University Suzhou 215123 Jiangsu People's Republic of China jplang@suda.edu.cn nkniuzheng@163.com +86-512-65880328; State Key Laboratory of Organometallic Chemistry, Shanghai Institute of Organic Chemistry, Chinese Academy of Sciences Shanghai 200032 People's Republic of China; School of Chemistry and Materials Engineering, Changshu Institute of Technology Changshu 215500 Jiangsu People's Republic of China lifeilong@cslg.cn

## Abstract

As an important two-dimensional material, layered double hydroxides (LDHs) show considerable potential in electrocatalytic reactions. However, the great thickness of the bulk LDH materials significantly limits their catalytic activity. In this work, we report ultrathin NiFe-LDH nanosheets with sulfate interlayer anions (Ni_6_Fe_2_(SO_4_)(OH)_16_·7H_2_O) (U-LDH(SO_4_^2−^)), which can be synthesized in gram-scale by a simple solvothermal method. The U-LDH(SO_4_^2−^) shows excellent stability and great electrocatalytic performance in OER with a current density of 10 mA cm^−2^ at a low overpotential of 212 mV and a small Tafel slope of 65.2 mV dec^−1^, exhibiting its great potential for a highly efficient OER electrocatalyst.

## Abstract 2

Ultrathin NiFe-LDH nanosheets with sulfate interlayer anions prepared in gram-scale *via* one-pot solvothermal method exhibited excellent OER activity.

## Graphical abstract

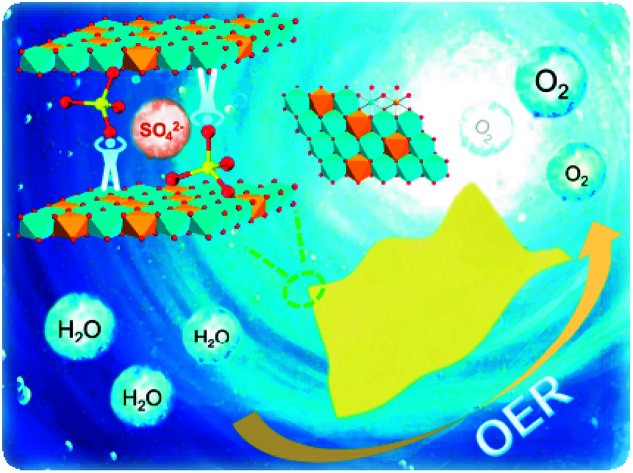

## Introduction

Current energy issues require the development of increasingly efficient and environmentally friendly energy conversion and storing devices.^1,2^ Water splitting, which includes the hydrogen evolution reaction (HER) and oxygen evolution reaction (OER), is considered to be a most promising approach for energy production.^3–6^ However, the sluggish kinetics of OER concerning multistep proton-coupled electron transfer processes has become a challenging topic and hampers efficiency of the electrocatalytic water splitting.^7–9^ Although noble-metal-based electrocatalysts (*e.g.* IrO_2_ and RuO_2_) possess high OER performance,^10^ the scarcity and high cost of such catalysts greatly hinder their industrial applications.

Layered double hydroxides (LDHs) with unique two-dimensional (2D) lamellar structures have been extensively investigated as OER electrocatalysts.^11–13^ However, the pristine LDHs show unsatisfactory performances in OER owing to their limited active sites and poor intrinsic activities.^11,14,15^ To date, more efforts have been devoted to improve the OER activity of LDH materials, including the incorporation of high-valent metals (such as V,^16^ Mn,^17^ Cr^18^), the fabrication of vacancies and defects,^19^ and the hybridization with highly conductive carbon materials.^20^ Since Hu and co-worker reported that monolayer LDH nanosheets exhibited high OER activity,^21^ the development of ultrathin LDH nanosheets for electrocatalysis has attracted great attention. Compared to the bulk LDHs, ultrathin LDH nanosheets with a thickness of a few nanometers have abundant active sites as well as the rapid mass transport and the superior electron transfer ability, thereby greatly enhancing catalytic performances.^21–24^ Exfoliation technology including liquid exfoliation^25,26^ and dry exfoliation^27^ is the most common method to prepare ultrathin nanosheet materials, but it is time-consuming, costly and low-yielding. Meanwhile, the restacking from the ultrathin LDH nanosheets into bulk LDHs is difficult to prevent.^27–29^ In this regard, a bottom-up, high-yield, wet chemical synthesis for obtaining LDH nanosheets appears to be a most promising strategy with potential practical applications.^30^ However, nanosheets prepared by conventional hydrothermal methods are usually thick and depend on the use of substrates (*e.g.* nickel foam (NF), carbon nanotubes, carbon paper),^31–33^ which limits their mass production and applications. Therefore, the facile and efficient large-scale synthesis of ultrathin nanosheets with uniform morphology represents a highly challenging target.

Few studies have shown that sulfate-intercalated LDH materials can be prepared directly under solvothermal conditions,^34,35^ because compared with other anions, CO_3_^2−^ get intercalated preferentially between the LDH layers, and they are stable and readily available, either from CO_2_ in ambient air or from certain synthetic precursors such as urea.^35^ Furthermore, the intrinsic characteristics of interlayer anions also have a certain effect on the electrocatalytic activity, such as the reducing ability, the chain length and the p*K*_a_ of the conjugate acid of the interlayer anions. However, small molecular interlayer anions such as SO_4_^2−^ show little effect on enhancing the intrinsic activity of LDH materials.^35–38^ Herein, we used a solvothermal method to directly prepare ultrathin NiFe-LDH nanosheets with sulfate interlayer anions (Ni_6_Fe_2_(SO_4_)(OH)_16_·7H_2_O) (U-LDH(SO_4_^2−^)), which can also be applied to its gram-scale synthesis. Different from the previous approaches of synthesizing only thicker nanosheets,^34,35^ this method used nickel acetate and ferrous sulfate as the raw materials for preparing ultrathin nanosheets, thus saving the time and reducing the cost. The obtained ultrathin NiFe-LDH nanosheets with a thickness of only a few atomic layers, exhibited higher electrocatalytic performance and durability toward OER than the commercial Ir/C in alkaline media and is superior to conventional NiFe-LDHs. The optimized U-LDH(SO_4_^2−^) achieved a current density of 10 mA cm^−2^ at a low overpotential of 212 mV with a small Tafel slope of 65.2 mV dec^−1^, and exhibited a high stability without significant activity decay for at least 11 h.

## Experimental section

### Materials

Nickel(ii) acetate tetrahydrate (Ni(OAc)_2_·4H_2_O, ≥ 98.0%), iron(ii) sulfate heptahydrate (FeSO_4_·7H_2_O, 99.0–101.0%), nickel(ii) nitrate hexahydrate (Ni(NO_3_)_2_·6H_2_O, 98%), iron(iii) nitrate nonahydrate (Fe(NO_3_)_3_·9H_2_O, ≥ 98.5%), ammonium fluoride (NH_4_F, ≥96.0%), urea (CH_4_N_2_O, 99%) and *N*,*N*-dimethylacetamide (DMAC) were purchased from Sinopharm Chemical Reagent Co. Ltd (Shanghai, China). All the chemicals were used without further purification.

### Preparation of U-LDH(SO_4_^2−^) nanosheets

Ni(OAc)_2_·4H_2_O (0.0249 g, 0.1 mmol) and FeSO_4_·7H_2_O (0.0084 g, 0.03 mmol) were each dissolved in 3 mL of H_2_O and their mixture was transferred into a Teflon-lined stainless steel autoclave followed by addition of 6 mL of DMAC. Subsequently, the reactor was sealed and placed upright in an oven and heated at 150 °C for 3 h. The as-prepared product was collected *via* centrifugation, washed with ethanol and water and dried at 60 °C for 12 h.

### Scale-gram preparation of U-LDH(SO_4_^2−^) nanosheets

An analogous procedure as the above reaction was used by using Ni(OAc)_2_·4H_2_O (2.4884 g, 10 mmol) and FeSO_4_·7H_2_O (0.8340 g, 3 mmol) in a mixed solvent of 600 mL DMAC : H_2_O = 1 : 1 (v/v) in a 1 L glass bottle.

### Preparation of B-LDH(CO_3_^2−^)

In a typical procedure^33^ but without the use of a Ni foam, Ni(NO_3_)_2_·6H_2_O (0.9887 g, 3.4 mmol), Fe(NO_3_)_3_·9H_2_O (0.6060 g, 1.5 mmol), NH_4_F (0.3700 g, 10 mmol), and urea (0.7500 g, 12.5 mmol) were dissolved in 70 mL deionized water and stirred for 30 min. The solution was transferred into a 100 mL of Teflon-lined stainless steel autoclave. The autoclave was heated at 120 °C for 6 h and naturally cooled down to room temperature. The as-obtained product was collected *via* centrifugation, washed with deionized water and ethanol and collected after being dried at 60 °C for 12 h.

### Preparation of Ni(OH)_2_

Ni(OAc)_2_·4H_2_O (0.0249 g, 0.1 mmol) dissolved in 12 mL mixed solvent (H_2_O : DMAC = 1 : 1 (v/v)). Then the reactor was sealed and placed upright in an oven and heated at 150 °C for 3 h. The as-obtained product was collected *via* centrifugation, washed with ethanol and water and dried at 60 °C for 12 h.

### Characterization

Scanning electron microscopy (SEM) images and energy dispersive X-ray spectroscopy (EDS) were taken at 15 kV by a HITACHI S-4700 cold field emission scanning electron microscope. Transmission electron microscopy (TEM) was performed on a FEI Tecnai G20 transmission electron microscope at an accelerating voltage of 200 kV. High-resolution TEM (HRTEM), high-angle annular dark-field scanning TEM (HAADF-STEM) and HAADF-STEM energy dispersive X-ray spectroscopy (HAADF-STEM-EDS) were performed on a FEI Tecnai F20 transmission electron microscope. The powder X-ray diffraction (PXRD) patterns were collected on X'Pert-Pro MPD diffractometer (Netherlands PANalytical) with a Cu Kα X-ray source (*λ* = 1.540598 Å). The thickness was determined by atomic force microscopy (AFM) using a Dimension-Icon (Bruker). The contents of Ni and Fe were measured by inductively coupled plasma atomic emission spectroscopy (ICP-AES) using a Varian 710-ES instrument (USA). X-ray photoelectron spectra (XPS) were collected on an SSI S-Probe XPS Spectrometer. FT-IR spectra (KBr pellets) were obtained with a Thermo Electron NEXUS 670 FT-IR spectrometer.

### Electrochemical measurements

The electrochemical measurements were carried out on an electrochemical working station (CHI 660E, Shanghai Chenhua). A three-electrode system was used with a saturated Ag/AgCl electrode as the reference and a Pt wire counter electrode, the working electrode was a glassy carbon electrode (GCE) (5 mm in diameter). To prepare the catalyst suspension, 2.5 mg of product and 2.5 mg of carbon powder were dispersed in 0.5 mL solution containing 485 μL isopropanol and 15 μL 0.5 wt% Nafion solution with the assistance of sonication for 1 h. Then, a 10 μL suspension was dropped onto the polished GCE and dried at room temperature. The potentials were referenced to the reversible hydrogen electrode (RHE) by the equation: *E*_RHE_ = *E*_Ag/AgCl_ + 0.197 + 0.059 pH and the overpotential (*η*) was calculated by the formula: *η* = *E*_RHE_ − 1.23 V. The polarization curves and Tafel plots were recorded in an O_2_-saturated 1.0 M KOH solution at a scan rate of 5 mV s^−1^ at room temperature and were iR-corrected. The stability test was performed by chronopotentiometry at a current density of 10 mA cm^−2^ without iR-compensation. The *C*_dl_ was determined from cyclic voltammograms measured in a non-faradaic region at different scan rates (*v* = 10, 20, 30, 40 and 50 mV s^−1^) in the potential range of 0.2 to 0.3 V *versus* Ag/AgCl. The electrochemically active surface area (ECSA) was estimated from the electrochemical double layer capacitance (*C*_dl_). Electrochemical impedance spectra (EIS) were measured with the sinusoidal wave amplitude of 10 mV and the frequency scan range of 10 to 0.1 kHz.

## Results and discussion

**Scheme 1:**
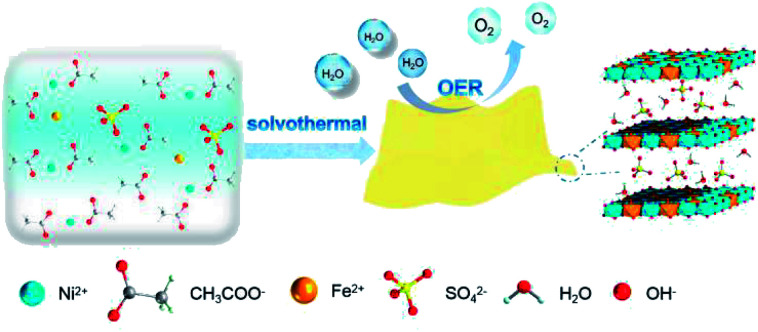
Synthetic procedure for U-LDH(SO_4_^2−^) nanosheets and its application in oxygen evolution reaction (OER).

U-LDH(SO_4_^2−^) was prepared *via* a one-pot solvothermal reaction (Scheme 1) using Ni(OAc)_2_·4H_2_O and FeSO_4_·7H_2_O (molar ratio = 10 : 3) as the metal sources, *N*,*N*-dimethylacetamide (DMAC) and H_2_O as a mixed solvent system, without the need for external alkali source or chelating reagent, which avoided the introduction of CO_3_^2−^ or other impurities, and saved the step of adjusting the pH value. At the elevated temperatures, the gradual hydrolysis of the acetate anions and the release of OH^−^ ions ensured a slow reaction between metal ions with OH^−^, thereby controlling nuclei formation and subsequent crystal growth to allow uniform precipitation.^39^ Sulfate plays a key role in the formation of ultrathin nanosheets. The size of the intercalated tetrahedral SO_4_^2−^ allowed an increase of the interlayer spacing compared to that of CO_3_^2−^. The larger interlayer spacing created by the sulfate anions weakened the interlayer interactions and facilitated further stripping.^40^ Meanwhile, water can act as a stripping agent at high temperatures.^30^ Using a 1 : 1 (v/v) DMAC : water mixture as the solvent system can not only reduce the solubility for carbonates, but also adjust the morphology of the target product (Fig. S1 and S2, ESI†). Hence, ultrathin sulfate-intercalated NiFe-LDH nanosheets can be successfully prepared. A possible formation mechanism can be proposed as follows:CH_3_COO^−^ + H_2_O → CH_3_COOH + OH^−^4Fe^2+^ + 2H_2_O + O_2_ → 4Fe^3+^ + 4OH^−^6Ni^2+^ + 2Fe^3+^ + 16OH^−^ + SO_4_^2−^ + 7H_2_O → Ni_6_Fe_2_(SO_4_)(OH)_16_·7H_2_O

**Fig. 1:**
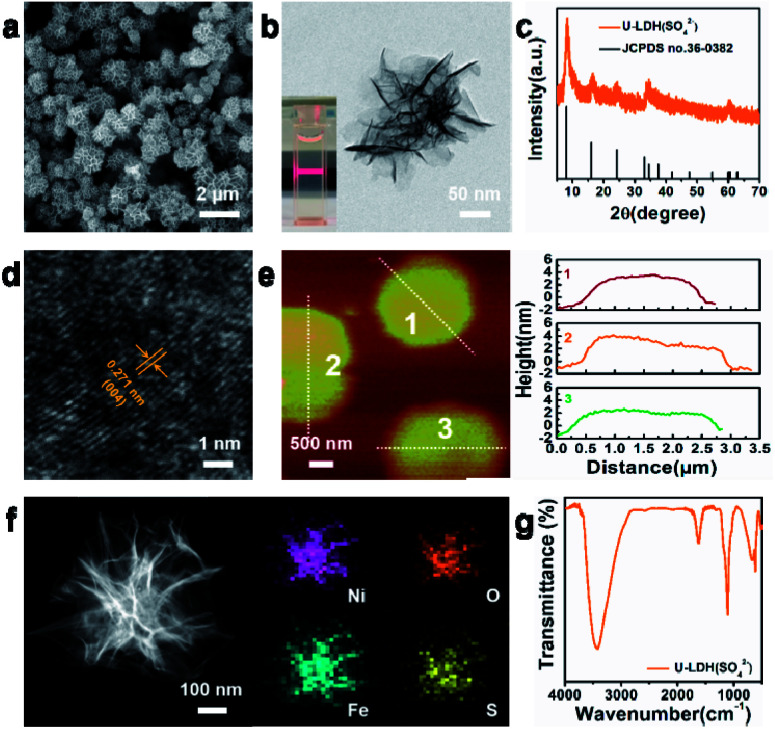
(a) SEM image, (b) TEM image, (c) PXRD patterns, (d) HRTEM image, (e) AFM image and height profiles, (f) HAADF-STEM image and EDX elemental mappings, (g) FT-IR spectrum of the U-LDH(SO_4_^2−^). The inset in (b) shows the Tyndall light scattering of the U-LDH(SO_4_^2−^) in aqueous solution.

The physical and structural characterization of U-LDH(SO_4_^2−^) are presented in Fig. 1. The 2D ultrathin morphology of U-LDH(SO_4_^2−^) showing uniform and flexible nanosheets was established by scanning electron microscopy (SEM) and transmission electron microscopy (TEM) (Fig. 1a and b). The Tyndall effect showed their good dispersibility in aqueous solution (inset of Fig. 1b). The powder X-ray diffraction (PXRD) patterns matched those of the natural mineral hydrohonessite (Ni_6_Fe_2_(SO_4_)(OH)_16_·7H_2_O, JCPDS #36-0382), which is a hydrated, sulfate-containing hydrocalcite-like compound (Fig. 1c). The broadening of the diffraction peaks is consistent with a small crystallite size and stacking defects.^38,40,41^ The interlayer spacing was calculated as 1.12 nm, larger than the interlayer spacing (0.79 nm) generated by carbonate anions, which greatly reduced the charge transfer resistance and improved the exchange capability with OH^−^ in the OER experiment.^40^ According to the high resolution TEM (HRTEM), the lattice spacing of 0.271 nm corresponds to the (004) facet of Ni_6_Fe_2_(SO_4_)(OH)_16_·7H_2_O (Fig. 1d). Due to the poor crystallinity of U-LDH(SO_4_^2−^), the lattice fringes were not so clear and regular, which was consistent with the PXRD results. Intriguingly, it is reported that low crystalline or amorphous materials may have better electrochemical performances than crystalline materials.^40,42^ The thickness of U-LDH(SO_4_^2−^) was determined by atomic force microscopy (AFM) to be in the range from 3 nm to 5 nm, a thickness corresponding to only three or four coordination layers (Fig. 1e). The energy dispersive X-ray spectroscopy (EDS) (Fig. S3, ESI†) confirmed the elemental composition of Ni, Fe, S, O, and the molar ratio Ni/Fe was calculated to be 3/1. According to the inductively coupled plasma atomic emission spectroscopy (ICP-AES), U-LDH(SO_4_^2−^) contains 34.2 wt% Ni and 12.2 wt% Fe (Ni/Fe = 2.8/1), which is close to the theoretical value (36.8 wt% Ni, 11.7 wt% Fe). Moreover, the HAADF-STEM images and energy-dispersive X-ray (EDX) elemental mappings (Fig. 1f) demonstrated the homogeneous distribution of these elements throughout the entire U-LDH(SO_4_^2−^). In the FT-IR spectrum (Fig. 1g), the bands at 1108 cm^−1^ and 618 cm^−1^ are attributed to the *ν*_3_(SO_4_^2−^) and *ν*_4_(SO_4_^2−^) modes,^43^ the peak at 1617 cm^−1^ and the broad band at 3416 cm^−1^ belong to the *δ*(H_2_O) and *ν*(H_2_O) vibration modes, respectively.^35^ No appreciable interlayer carbonate was found in U-LDH(SO_4_^2−^).

**Fig. 2:**
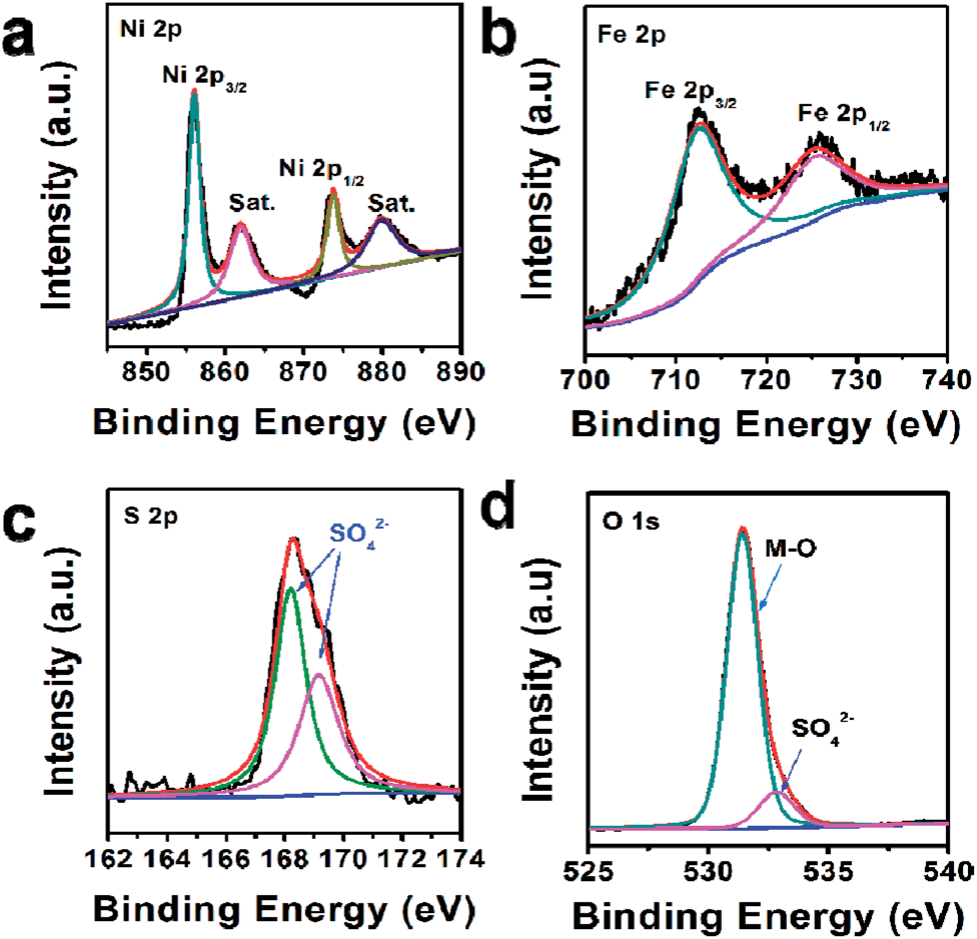
XPS spectra of U-LDH(SO_4_^2−^): (a) Ni 2p, (b) Fe 2p, (c) S 2p, (d) O 1s.

In addition, the composition of U-LDH(SO_4_^2−^) was further clarified by X-ray photoelectron spectroscopy (XPS). The survey spectra (Fig. 2) showed that U-LDH(SO_4_^2−^) is composed of Ni, Fe, O and S elements, which is consistent with the EDS and EDX mapping results. In the high-resolution Ni 2p spectrum (Fig. 2a), the peaks of Ni 2p_3/2_ and Ni 2p_1/2_ at 856.0 eV and 873.5 eV, along with two satellite peaks at 861.8 eV and 879.7 eV, indicating the valence state of +2 for the Ni. In the high-resolution Fe 2p spectrum, the binding energy peaks of Fe 2p_3/2_ and 2p_1/2_ are located at 712.3 eV and 725.2 eV, respectively, demonstrating Fe^2+^ ions were spontaneously oxidized to Fe^3+^ ions during the reaction (Fig. 2b). And the peaks at 168.1 eV and 169.3 eV in the S 2p spectrum indicate the presence of SO_4_^2−^ anions (Fig. 2c). As for O 1s spectrum, the binding energies at 531.4 eV and 532.8 eV are assignable to the M–O and SO_4_^2−^, respectively (Fig. 2d).

**Fig. 3:**
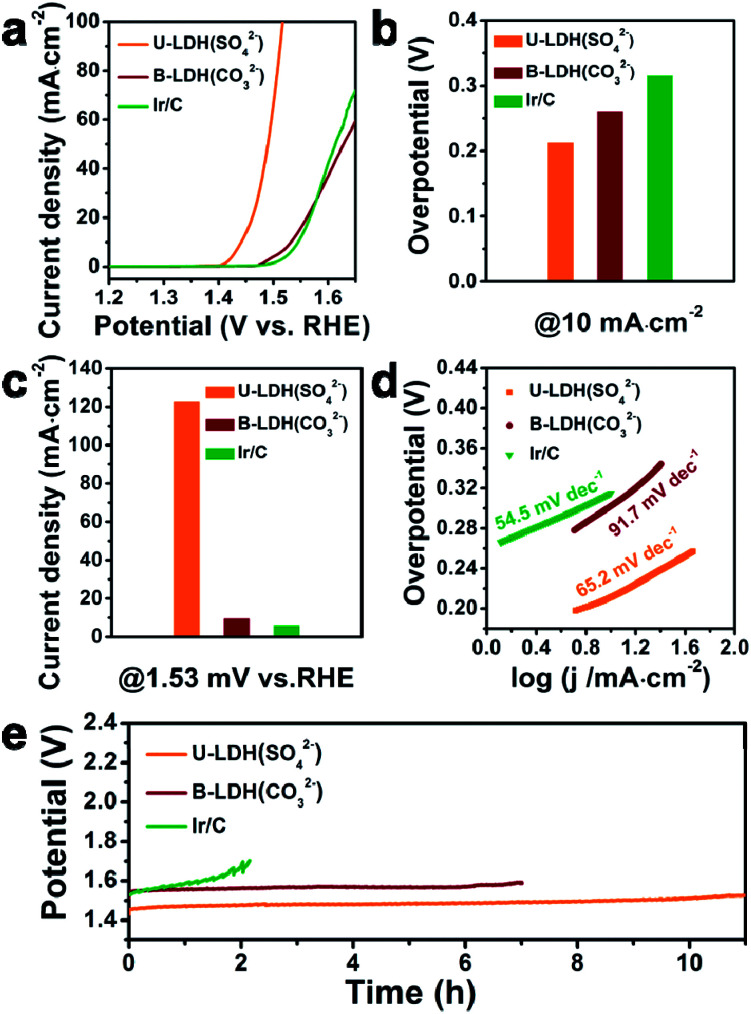
(a) Linear sweep voltammetry OER curves of U-LDH(SO_4_^2−^), B-LDH(CO_3_^2−^) and commercial Ir/C. (b) Overpotentials of different catalysts at the current density of 10 mA cm^−2^. (c) Current densities of different catalysts at 1.53 V *versus* RHE. (d) Tafel plots of different catalysts. (e) Chronopotentiometry tests of U-LDH(SO_4_^2−^), B-LDH(CO_3_^2−^) and commercial Ir/C in 1 M KOH at 10 mA cm^−2^.

For comparison, the bulk NiFe-LDH with carbonate interlayer anions (B-LDH(CO_3_^2−^)) (B stands for bulk) was prepared (Fig. S4–S6, ESI†). The PXRD patterns of the B-LDH(CO_3_^2−^) were in agreement with those of the reference NiFe-LDH (Ni_0.75_Fe_0.25_(CO_3_)_0.125_(OH)_2_·0.38H_2_O, JCPDS #40-0215) (Fig. S4c, ESI†). The U-LDH(SO_4_^2−^), B-LDH(CO_3_^2−^) and Ir/C were subjected to OER tests in 1 M KOH aqueous solution. In order to minimize capacitive current, a slow scan rate of 5 mV s^−1^ was used. Linear sweep voltammetry was employed to obtain their polarization curves (Fig. 3a). As demonstrated in Fig. 3b, U-LDH(SO_4_^2−^) exhibits excellent OER performance with an overpotential of 212 mV at a current density of 10 mA cm^−2^, much lower than those of the commercial Ir/C (315 mV) and B-LDH(CO_3_^2−^) (303 mV). Furthermore, at the potential of 1.53 V (*vs.* RHE), U-LDH(SO_4_^2−^), B-LDH(CO_3_^2−^) and Ir/C reached a current density of 122.75, 9.44 and 5.64 mA cm^−2^, respectively (Fig. 3c). The Tafel slope of U-LDH(SO_4_^2−^) (65.2 mV dec^−1^), slightly higher than that of Ir/C (54.5 mV dec^−1^) and much lower than that of B-LDH(CO_3_^2−^) (91.7 mV dec^−1^), reveals the fast OER kinetics obtained with U-LDH(SO_4_^2−^) (Fig. 3d). The stability of electrocatalyst material is also a significant criterion for evaluating the performance of a catalyst. U-LDH(SO_4_^2−^) showed favorable stability since the performance of U-LDH (SO_4_^2−^) can be maintained in 1 M KOH for 11 h, a much longer time than those showed by using Ir/C and B-LDH (CO_3_^2−^) (Fig. 3e). The enhanced durability of the U-LDH(SO_4_^2−^) is mainly ascribed to its ultrathin sheet morphology, so it can better adhere to the surface of glassy carbon electrode and reduce shedding during the OER test.^44^ All these pertinent electrocatalytic parameters exhibited by U-LDH(SO_4_^2−^) are better than those drived from the conventional NiFe-LDHs and the Fe/Ni-based catalysts (Table S1, ESI†).

**Fig. 4:**
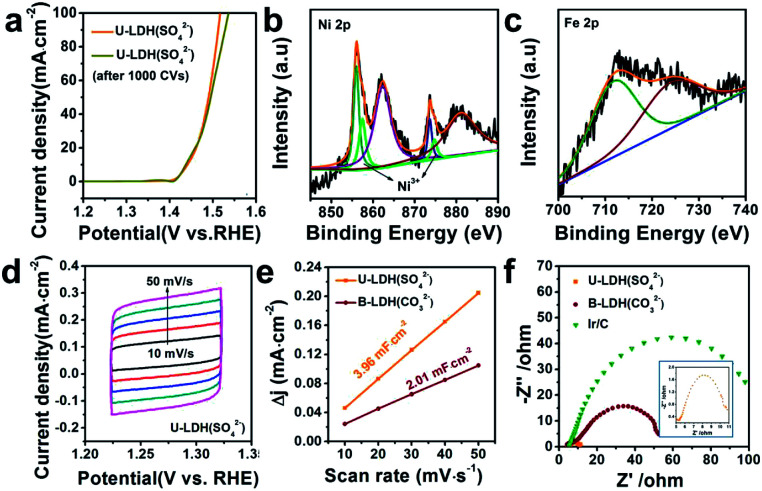
(a) Polarization curves of U-LDH(SO_4_^2−^) before and after 1000 CV cycles. XPS spectrum of (b) Ni 2p and (c) Fe 2p of U-LDH(SO_4_^2−^) after 1000 CV cycles. (d) CV curves in potential range of 1.22–1.32 V *versus* RHE of U-LDH(SO_4_^2−^). (e) Electrochemical double-layer capacitance (*C*_dl_) of U-LDH(SO_4_^2−^) and B-LDH(CO_3_^2−^). (f) EIS Nyquist plots of U-LDH(SO_4_^2−^), B-LDH(CO_3_^2−^) and Ir/C at 1.5 V *vs.* RHE in 1 M KOH.

The OER polarization curve of U-LDH(SO_4_^2−^) unveiled only minor changes after 1000 cyclic voltammetry (CV) cycles (Fig. 4a), further implying its good electrochemical stability for OER. TEM image of U-LDH(SO_4_^2−^) (Fig. S7, ESI†) revealed that its original morphology got almost retained after a long-term stability test. Moreover, XPS technique was applied to evaluate the elemental valence states of U-LDH(SO_4_^2−^) after the OER measurement (Fig. S8, ESI†). The high-resolution Ni 2p spectrum (Fig. 4b) contains new peaks of Ni 2p_3/2_ (857.4 eV) and Ni 2p_1/2_ (874.7 eV), indicating that NiOOH might be formed during the electrocatalytic process. The peaks of Fe 2p_3/2_ and Fe 2p_1/2_ at 711.8 eV and 724.1 eV are characteristic for the binding energy of Fe^3+^ in FeOOH, which is the critical active phase for OER (Fig. 4c).^45,46^ To further illustrate the OER mechanism of U-LDH(SO_4_^2−^), Ni(OH)_2_ was prepared by a method similar to that for U-LDH(SO_4_^2−^) (Fig. S9, ESI†). The cyclic voltammogram measurements of U-LDH(SO_4_^2−^) and Ni(OH)_2_ were carried out (Fig. S10, ESI†). As shown in the CV curves, Ni(OH)_2_ exhibited a quasi-reversible Ni^2+/3+^ redox behavior at *E*_1/2_ ≈ 1.35 V.^47^ As reported in previous literatures,^47,48^ the introduction of Fe led to a positive shift of this function, thus confirming the strong interactions between nickel and iron ions. In addition, the broad peak O^2^ at about 1.47 V was attributed to the presence of Ni^4+^ or Fe^4+^, which might make a significant contribution to the OER performance.^33,49,50^

Notably, both the number of exposed active sites (extrinsic) and unit activity on each active site (intrinsic) have a significant impact on OER performance. The electrochemical surface areas (ECSA) of various samples were obtained by cyclic voltammetry at various scan rates in the range of non-faradaic potential (Fig. 4d and S11, ESI†). The double layer capacitance (*C*_dl_) values were calculated by the plots of Δ*j* = (*j*_a_ − *j*_c_)/2 at 1.27 V *vs.* RHE against the scan rate (Fig. 4e). The *C*_dl_ of U-LDH(SO_4_^2−^), B-LDH(CO_3_^2−^) were 3.96 and 2.01 mF cm^−2^, respectively. Benefitting from the ultrathin sheets morphology with a thickness of only a few atomic layers, the *C*_dl_ value of U-LDH(SO_4_^2−^) was twice higher than that of B-LDH(CO_3_^2−^), indicating that U-LDH(SO_4_^2−^) owned a larger active surface area, which was responsible for the excellent OER activity.^21,51^ To investigate the electron transport capability of U-LDH(SO_4_^2−^), electrochemical impedance spectroscopy (EIS) was performed. As shown in the Nyquist plots, the semicircular diameter of U-LDH(SO_4_^2−^) was significantly smaller than those of B-LDH(CO_3_^2−^) and Ir/C (Fig. 4f), suggesting its lower charge transfer impedance, the faster charge transfer and the higher electrical conductivity. Remarkably, a large-scale synthesis of U-LDH(SO_4_^2−^) can be realized in a 1 L reactor, yielding 1.438 g of product (Fig. S12, ESI†) with the same morphology and crystal structure (Fig. S13†), and also displaying the same excellent OER performance (Fig. S14, ESI†).

## Conclusions

In this work, we prepared an ultrathin sulfate-intercalated NiFe-LDH nanosheets U-LDH(SO_4_^2−^) in gram-scale by using a facile solvothermal reaction. This material showed excellent electrocatalytic performance in OER and could attain a current density of 10 mA cm^−2^ at a low overpotential of 212 mV, and maintained for at least 11 h without significant activity decay. In particular, the sulfate interlayer anions play a key role in the construction of the ultrathin nanosheets. Benefiting from the two-dimensional structure, ultrathin sheet morphology and bimetallic composition, U-LDH(SO_4_^2−^) held a large active surface area, a low charge transfer impedance, a low overpotential, a small Tafel slope and a superior stability, demonstrating that it would be an ideal electrocatalyst for OER. This work also provides some insight into the design and preparation of novel Ni/Fe(Co, Mn, *etc.*)-based LDHs and other 2D lamellar materials with excellent performances in OER and other electrocatalytic reactions.

## Conflicts of interest

There are no conflicts to declare.

## Supplementary Material

RA-010-D0RA00845A-s001
