# Supplementary material for: Ultrathin sulfate-intercalated NiFe-layered double hydroxide nanosheets for efficient electrocatalytic oxygen evolution
Source: RSC Adv. 2020 Mar 25;10(21):12145–50. doi: 10.1039/d0ra00845a (PMC9051218; doi:10.1039/d0ra00845a)
Supplement: RA-010-D0RA00845A-s001 [file RA-010-D0RA00845A-s001.pdf]

Electronic Supplementary Material (ESI) for RSC Advances.
This journal is © The Royal Society of Chemistry 2020

Electronic Supplementary Information

Ultrathin sulfate-intercalated NiFe-layered double hydroxides nanosheets for efficient electrocatalytic oxygen evolution

Xiao-Xiao Jiang,[a,b] Jiang-Yan Xue,[a] Zhong-Yin Zhao,[a] Cong Li,[a] Fei-Long Li,[c]* Chen Cao,[a] Zheng Niu,[a]* Hong-Wei Gu,[a] and Jian-Ping Lang[a,b]*

[a]*College of Chemistry, Chemical Engineering and Materials Science, Soochow University, Suzhou 215123, People's Republic of China*

[b]*State Key Laboratory of Organometallic Chemistry, Shanghai Institute of Organic Chemistry, Chinese Academy of Sciences, Shanghai 200032, People's Republic of China*

[c]*School of Chemistry and Materials Engineering, Changshu Institute of Technology, Changshu 215500, Jiangsu, People's Republic of China*

* Correspondence authors at: *College of Chemistry, Chemical Engineering and Materials Science, Soochow University, Suzhou 215123, People's Republic of China*.

Tel: +86-512-65882865; fax: +86-512-65880328.

E-mail address: jplang@suda.edu.cn (J.P. Lang).

Contents

Fig. S1 (a) TEM image and (b) PXRD patterns of the products obtained under similar conditions of U-LDH(SO_4^{2-}) except that only water was used as the reaction solvent……………………......…S3

Fig. S2 (a) TEM image and (b) PXRD patterns of the products obtained under similar conditions of U-LDH(SO_4^{2-}) except that only DMAC was used as the reaction solvent………………......….S3

Fig. S3 EDX spectrum of U-LDH(SO_4^{2-})……………………………………………….…...S4

Fig. S4 (a) SEM image, b) TEM image and (c) PXRD patterns of B-LDH(CO_3^{2-})……………..…...S4

Fig. S5. XPS spectra of B-LDH(CO_3^{2-}): (a) survey scan, (b) Ni 2p, (c) Fe 2p, (d) O 1s, (e) C 1s, (f) F 1s………………………………………………………………………………...….S5

Fig. S6 FT-IR spectra of U-LDH(SO_4^{2-}) and B-LDH(CO_3^{2-}).……………………...…………...…S5

Table S1 Comparisons of OER performance for Fe/Ni-based and LDH-based electrocatalysts in 1 M KOH. ………………………………………………………………………...………S6

Fig. S7 TEM images of U-LDH(SO_4^{2-}) after 1000 CV cycles. ……………………………...…S8

Fig. S8 XPS spectra of U-LDH(SO_4^{2-}) after 1000 CV cycles: (a) survey scan, (b) O 1s, (c) C 1s, (d) S 2p. ....……………………………………………………………………………….S8

Fig. S9 (a) SEM image, (b) TEM image, (c) PXRD patterns of $Ni(OH)_2$. ……………………….S9

Fig. S10 CV curves of $Ni(OH)_2$ and U-LDH(SO_4^{2-}) at a scan rate of 5 mV·s^{-1}…………...….…S9

Fig. S11 CV curves in a potential range of 1.22-1.23 V versus RHE of U-LDH(SO_4^{2-})…..…….S10

Fig. S12 Photograph of U-LDH(SO_4^{2-}) prepared in gram-scale: (a) U-LDH(SO_4^{2-}) prepared in a 1 L reactor, b) powder of U-LDH(SO_4^{2-}) weighed on a precision electronic balance…..............…S10

Fig. S13 (a, b) TEM images, (c) PXRD patterns of U-LDH(SO_4^{2-}) prepared in gram-scale…... S11

Fig. S14 (a) Linear sweep voltammetry OER curve and (b) Tafel plot of U-LDH(SO_4^{2-}) prepared in gram-scale…..……………………………………………………….……...…..S11

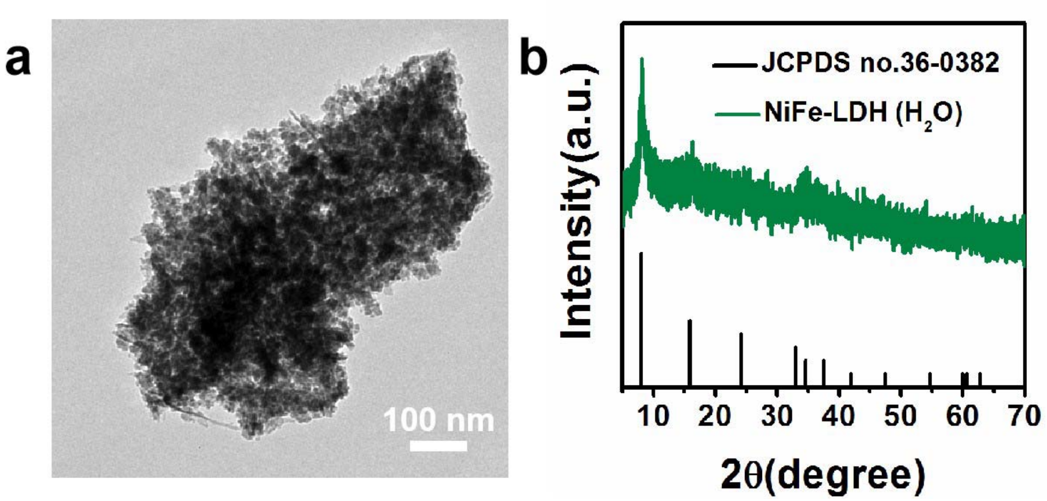

Fig. S1 (a) TEM image and (b) PXRD patterns of the products obtained under similar conditions of U-LDH(SO_4^{2-}) except that only water was used as the reaction solvent.

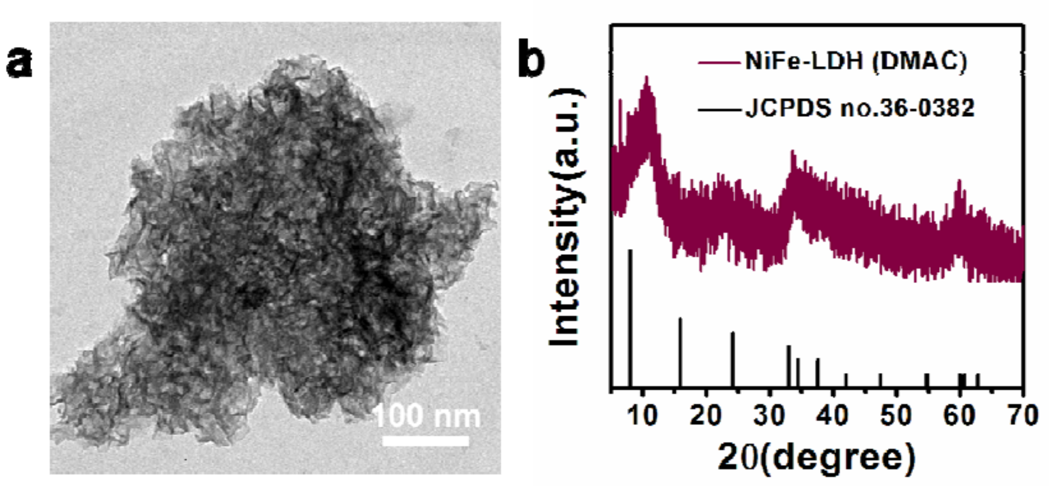

Fig. S2 (a) TEM image and (b) PXRD patterns of the products obtained under similar conditions of U-LDH(SO_4^{2-}) except that only DMAC was used as the reaction solvent.

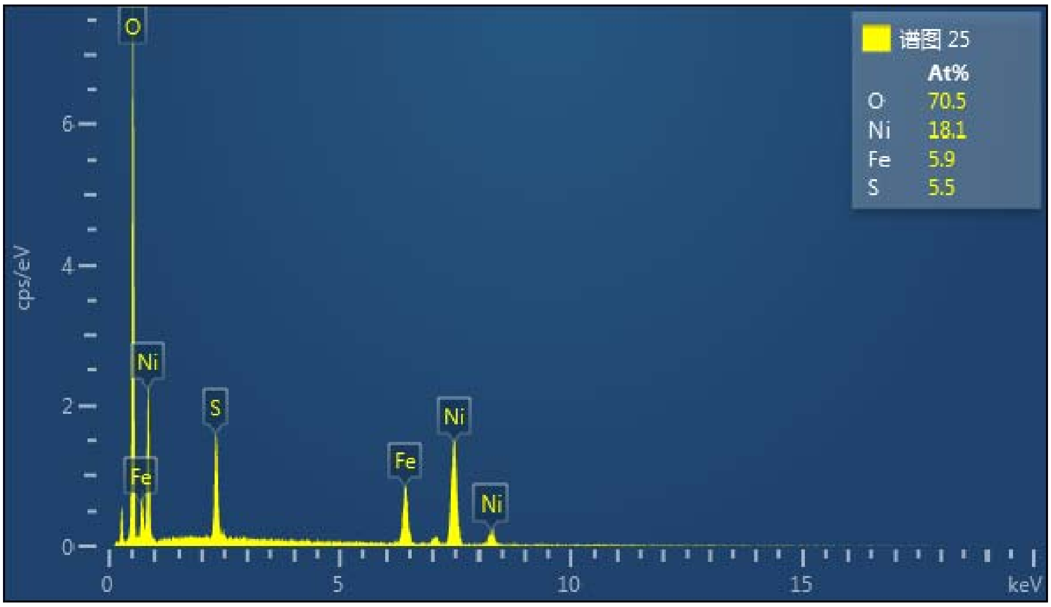

Fig. S3 EDX spectrum of U-LDH(SO_4^{2-}).

Noted: (**n(Ni) : n(Fe) : n(S)** = 18.1: 5.9: 5.5 = 3: 1: 0.93)

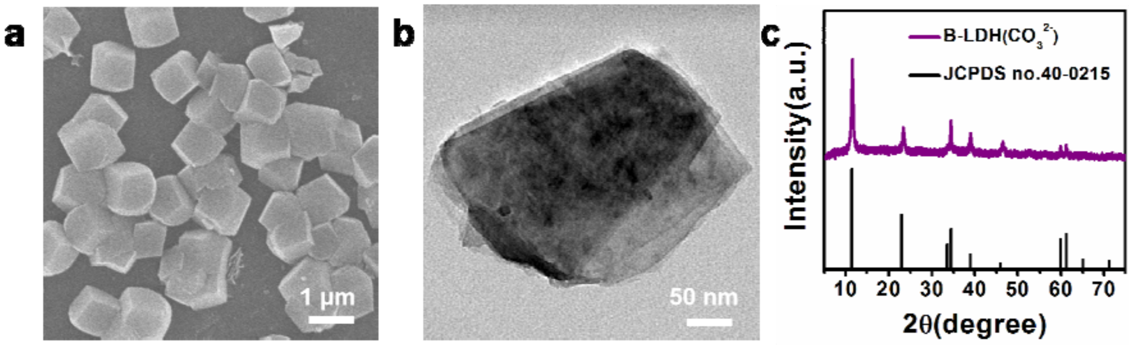

Fig. S4 (a) SEM image, (b) TEM image and (c) PXRD patterns of B-LDH(CO_3^{2-}).

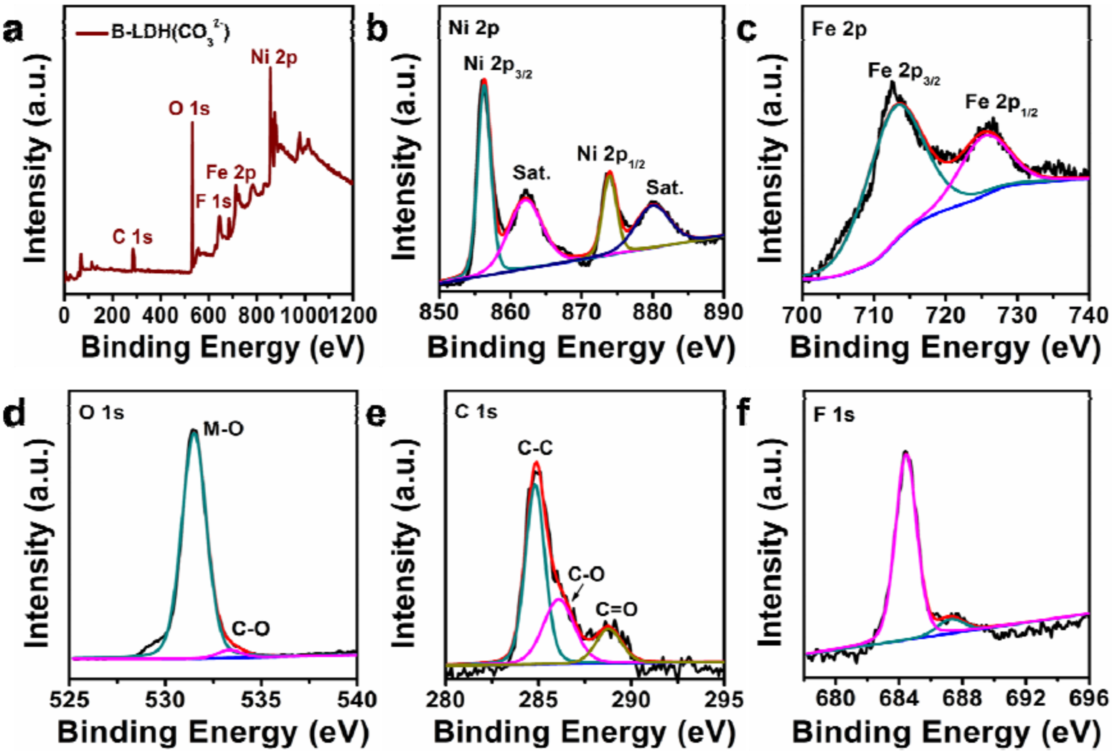

Fig. S5 XPS spectra of B-LDH(CO_3^{2-}): (a) survey scan, (b) Ni 2p, (c) Fe 2p, (d) O 1s, (e) C 1s, (f) F 1s.

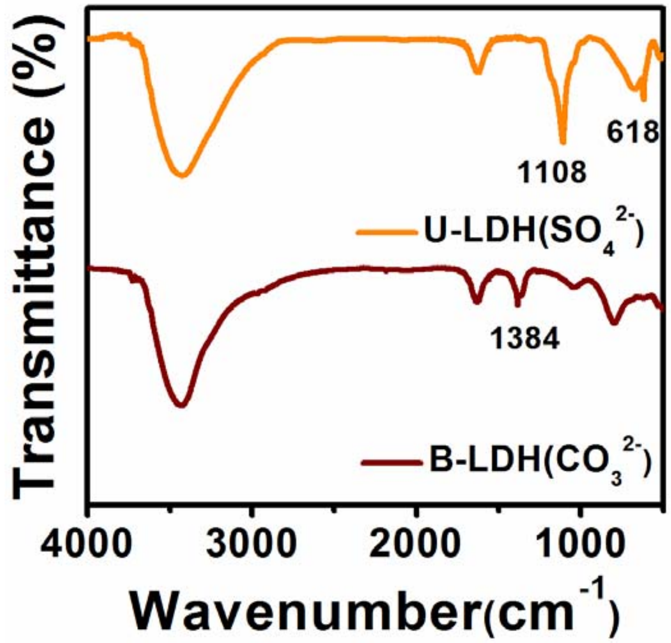

Fig. S6 FT-IR spectra of U-LDH(SO_4^{2-}) and B-LDH(CO_3^{2-}).

Noted: The peak at 1384 cm^{-1} is attributed to $v_3(CO_3^{2-})$, while those at 1108 cm^{-1} and 618 cm^{-1} are attributed to the $v_3(SO_4^{2-})$ and $v_4(SO_4^{2-})$.

Table S1 Comparisons of OER performance for Fe/Ni-based and LDH-based electrocatalysts in 1 M KOH.

**Catalysts**	**Electrode**	**Overpotential (mV) at 10 mA·cm^{-2}**	**Tafel slope (mV·dec^{-1})**	**Reference**
U-LDH(SO_4^{2-})	GCE	212	65.2	**This work**
SO_4^{2-} (EG) NiFe LDH	Carbon paper	375	56	*Chem. Mater.*, 2018, **30**, 4321-4330.
NiFe-SO_4	Ni foam	356	93	*Chem. Mater.*, 2019, **31**, 6798-6807.
Ni-Fe LDH nanoprisms	GCE	280	49.4	*Angew. Chem. Int. Ed.,* 2018, **57**, 172-176.
Ni/NiO@CoFe LDH	Ni/NiO foam	230	34.3	*ChemSusChem,* 2019, **12**, 2773-2779.
NiFe/Cu_2O NWs/CF	Cu foam	284	42	*ChemSusChem,* 2017, **10,** 1475-1481.
$Fe(OH)_3$@Co-MOF-74	carbon paper	292	44	*ChemSusChem,* 2019, **12**, 4623-4628.
NiFe-LDH-UF (UF: Ultrafine)	Graphite paper	254	32	*Adv. Energy Mater.,* 2018, **8**, 1703585.
NiFe hydroxide	GCE	270	36.2	*Angew. Chem. Int. Ed.*, 2019, **58**, 736-740.
δ-FeOOH NSs/NF	Ni foam	265	36	*Adv. Mater.,* 2018, **30**, 1803144.
CoMn-LDH	GCE	325	43	*J. Am. Chem. Soc.,* 2014, **136** , 16481-16484.
NiFeRu LDH/Ni foam	Ni foam	225	32.4	*Adv. Mater.*, 2018, **30**,

				1706279.
$Ni_{0.75}Fe_{0.125}V_{0.125}$-LDHs/ NF	Ni foam	231	39.4	*Small,* 2018, **14**, 1703257.
Cu@CoFe LDH	Cu foam	240	44.4	*Nano Energy,* 2017, **41**, 327-336.
CoFe LDHs-Ar	GCE	266	37.85	*Angew. Chem. Int. Ed.,* 2017, **56**, 5867-5871.

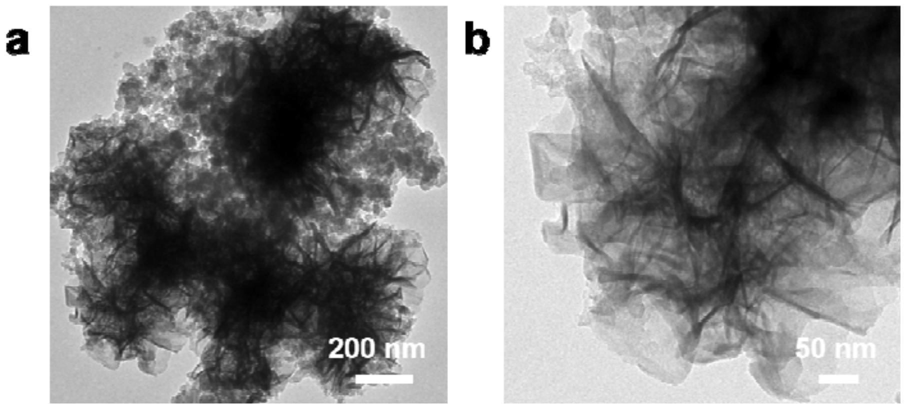

Fig. S7 TEM images of U-LDH(SO_4^{2-}) after 1000 CV cycles.

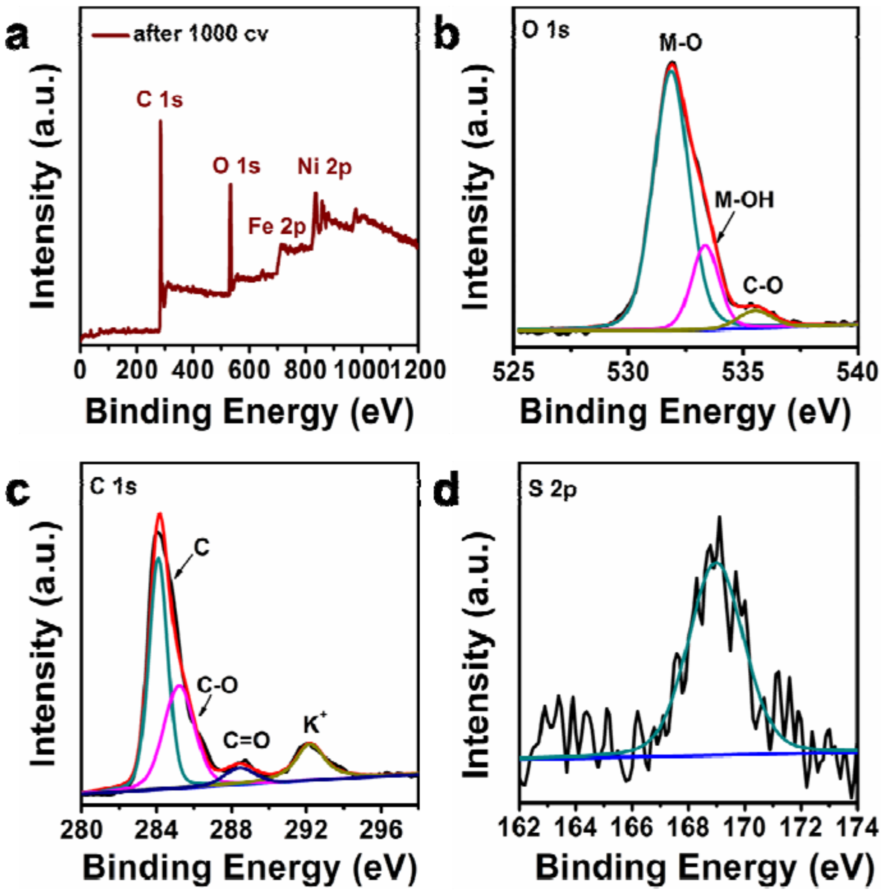

Fig. S8 XPS spectra of U-LDH(SO_4^{2-}) after 1000 CV cycles: (a) survey scan, (b) O 1s, (c) C 1s, (d) S 2p.

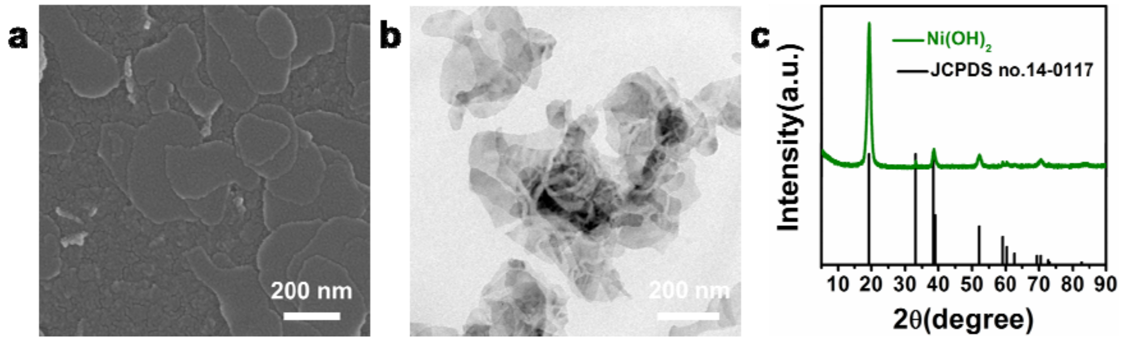

Fig. S9 (a) SEM image, (b) TEM image, (c) PXRD patterns of $Ni(OH)_2$.

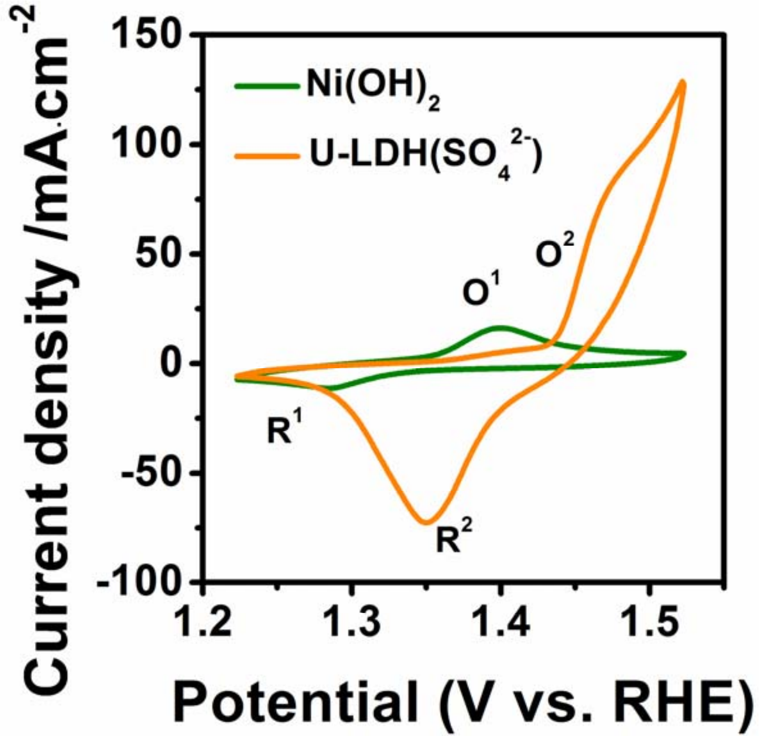

Fig. S10 CV curves of $Ni(OH)_2$ and U-LDH(SO_4^{2-}) at a scan rate of 5 $mV\cdot s^{-1}$.

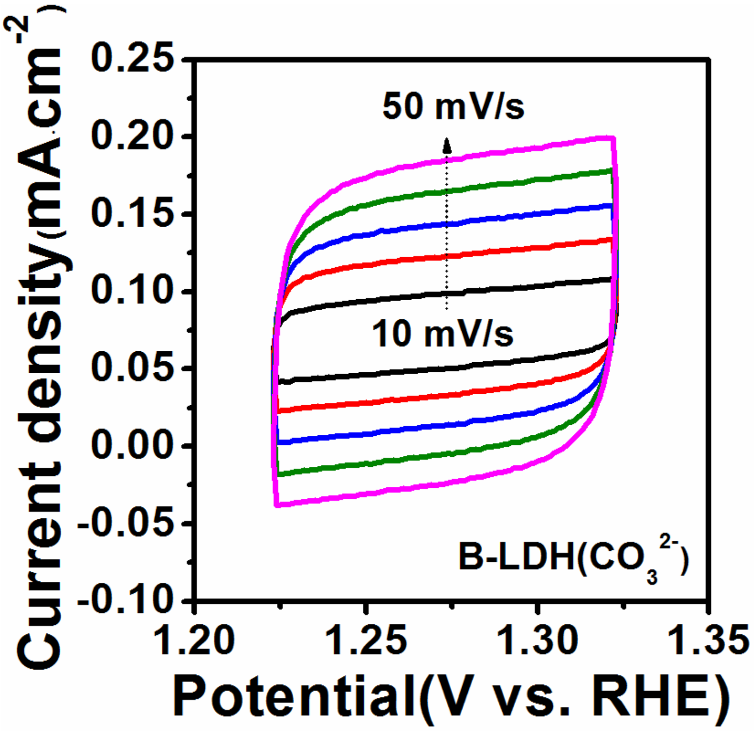

Fig. S11 CV curves in a potential range of 1.22-1.23 V versus RHE of U-LDH(SO_4^{2-}).

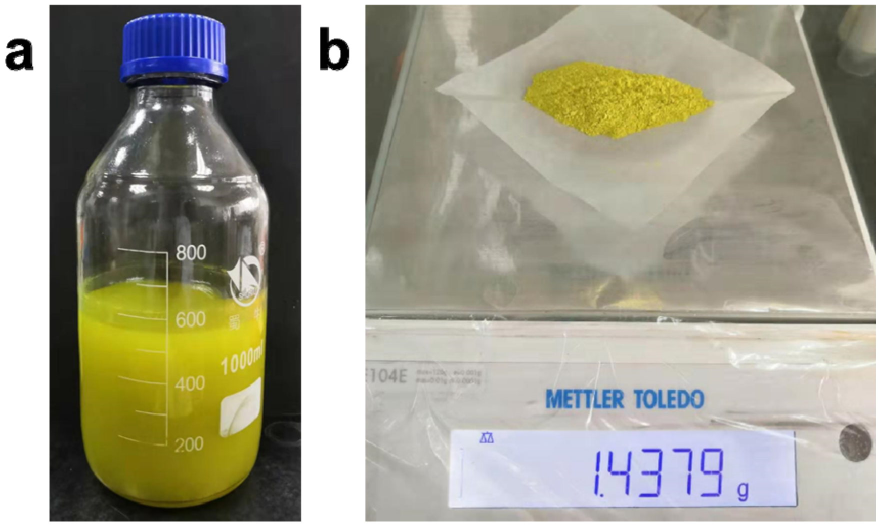

Fig. S12 Photograph of U-LDH(SO_4^{2-}) prepared in gram-scale: (a) U-LDH(SO_4^{2-}) prepared in a 1 L reactor, (b) powder of U-LDH(SO_4^{2-}) weighed on a precision electronic balance.

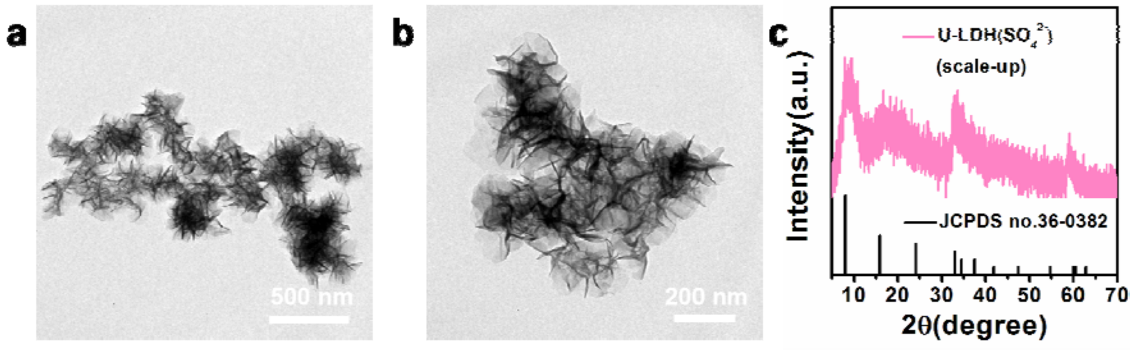

Fig. S13 (a, b) TEM images, (c) PXRD patterns of U-LDH(SO_4^{2-}) prepared in gram-scale.

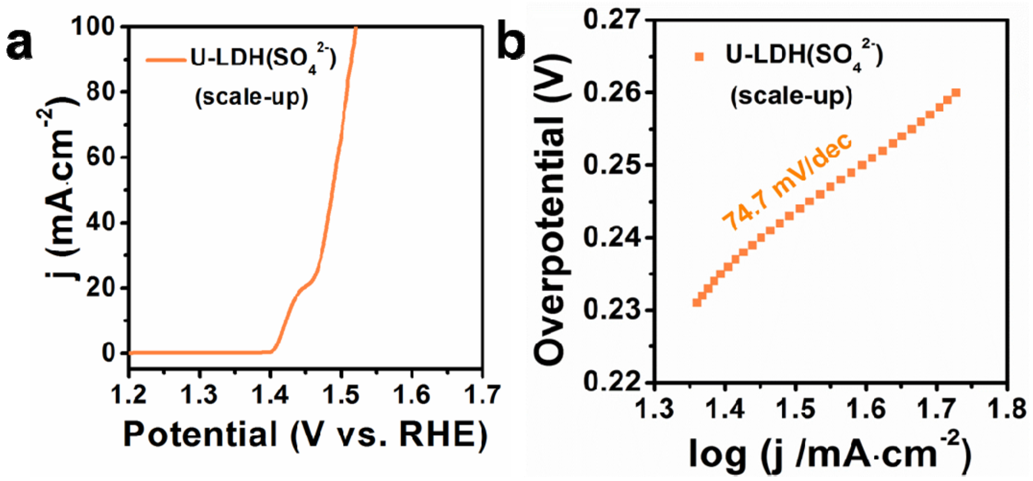

Fig. S14 (a) Linear sweep voltammetry OER curve and (b) Tafel plot of U-LDH(SO_4^{2-}) prepared in gram-scale.
